# Attention to the Variation of Probabilistic Events: Information Processing with Message Importance Measure

**DOI:** 10.3390/e21050439

**Published:** 2019-04-26

**Authors:** Rui She, Shanyun Liu, Pingyi Fan

**Affiliations:** Beijing National Research Center for Information Science and Technology, Department of Electronic Engineering, Tsinghua University, Beijing 100084, China

**Keywords:** message importance measure, information theory, probabilistic events processing, message transmission and compression

## Abstract

Different probabilities of events attract different attention in many scenarios such as anomaly detection and security systems. To characterize the events’ importance from a probabilistic perspective, the message importance measure (MIM) is proposed as a kind of semantics analysis tool. Similar to Shannon entropy, the MIM has its special function in information representation, in which the parameter of MIM plays a vital role. Actually, the parameter dominates the properties of MIM, based on which the MIM has three work regions where this measure can be used flexibly for different goals. When the parameter is positive but not large enough, the MIM not only provides a new viewpoint for information processing but also has some similarities with Shannon entropy in the information compression and transmission. In this regard, this paper first constructs a system model with message importance measure and proposes the message importance loss to enrich the information processing strategies. Moreover, the message importance loss capacity is proposed to measure the information importance harvest in a transmission. Furthermore, the message importance distortion function is discussed to give an upper bound of information compression based on the MIM. Additionally, the bitrate transmission constrained by the message importance loss is investigated to broaden the scope for Shannon information theory.

## 1. Introduction

In recent years, massive data has attracted much attention in various realistic scenarios. Actually, there exist many challenges for data processing such as distributed data acquisition, huge-scale data storage and transmission, as well as correlation or causality representation [1,2,3,4,5]. Facing these obstacles, it is a promising way to make good use of information theory and statistics to deal with mass information. For example, a method based on Max Entropy in Metric Space (MEMS) is utilized for local features extraction and mechanical system analysis [6]; as an information measure different from Shannon entropy, Voronoi entropy is discussed to characterize the random 2D patterns [7]; Category theory, which can characterize the Kolmogorov–Sinai and Shannon entropy as the unique functors, is used in autonomous and networked dynamical systems [8].

To some degree, probabilistic events attract different interests according to their probability. For example, considering that small probability events hidden in massive data contain more semantic importance [9,10,11,12,13], people usually pay more attention to the rare events (rather than the common events) and design the corresponding strategies of their information representation and processing in many applications including outliers detection in the Internet of Things (IoT), smart cities and autonomous driving [14,15,16,17,18,19,20,21,22]. Therefore, the probabilistic events processing has special values in the information technology based on semantics analysis of message importance.

In order to characterize the importance of probabilistic events, a new information measure named MIM is presented to generalize Shannon information theory [23,24,25]. Here, we shall investigate the information processing including compression (or storage) and transmission based on MIM to bring some new viewpoints in the information theory. Now, we first give a short review on MIM.

### 1.1. Review of Message Importance Measure

Essentially, the message importance measure (MIM) is proposed to focus on the probabilistic events importance [23]. In particular, the core idea of this information measure is that the weights of importance are allocated to different events according to the corresponding events’ probability. In this regard, as an information measure, MIM may provide an applicable criterion to characterize the message importance from the viewpoint of inherent property of events without the human subjective factors. For convenience of calculation, an exponential expression of MIM is defined as follows.

**Definition** **1.** *For a discrete distribution P(X) = $\{p\left(x_{1}\right)$, $p(x_{2})$, …, $p(x_{n})\}$, the exponential expression of message importance measure (MIM) is given by*

(1) $$\begin{array}{cc} L(\varpi ,X) & =\sum_{x_{i}}p\left(x_{i}\right)e^{\varpi \{1-p\left(x_{i}\right)\}}, \end{array}$$

*where the adjustable parameter ϖ is nonnegative and $p\left(x_{i}\right)e^{\varpi \{1-p\left(x_{i}\right)\}}$ is viewed as the self-scoring value of event i to measure its message importance.*

Actually, from the perspective of generalized Fadeev’s postulates, the MIM is viewed as a rational information measure similar to Shannon entropy and Renyi entropy which are respectively defined by

(2a) $$\begin{array}{cc} & H\left(X\right)=-\sum_{x_{i}}p\left(x_{i}\right)\log p(x_{i}), \end{array}$$

(2b) $$\begin{array}{cc} & H_{\alpha }\left(X\right)=\frac{1}{1-\alpha }\log\sum_{x_{i}}{\left\{p\left(x_{i}\right)\right\}}^{\alpha },\;\left(0<\alpha <\infty ,\alpha \neq 1\right), \end{array}$$

where the condition of variable *X* is the same as that described in Definition 1. In particular, a postulate for the MIM weaker than that for Shannon entropy and Renyi entropy is given by

(3) F(PQ)≤F(P)+F(Q),

while F(PQ)=F(P)+F(Q) is satisfied for Shannon entropy and Renyi entropy [26], where *P* and *Q* are two independent random distributions and F(·) denotes a kind of information measure.

Moreover, the crucial operator of MIM to handle probability elements is exponential function while the corresponding operators of Shannon and Renyi entropy are logarithmic function and polynomial function respectively. In this case, MIM can be viewed as a map for the assignments of events’ importance weights or the achievement for the self-scoring values of events different from conventional information measures.

As far as the application of MIM is concerned, it may be a better method by using this information measure to detect unbalanced events in signal processing. Ref. [27] has investigated the minor probability event detection by combining MIM and Bayes detection. Moreover, it is worth noting that the physical meaning of the components of MIM corresponds to the normalized optimal data recommendation distribution, which makes a trade-off between the users’ preference and system revenue [28]. In this respect, MIM plays a fundamental role in the recommendation system (a popular applications of big data) from the theoretic viewpoint. Therefore, MIM does not come from the imagination directly, whereas it is a meaningful information measure originated from the practical scenario.

### 1.2. The Importance Coefficient ϖ in MIM

In general, the parameter ϖ viewed as the importance coefficient has a great impact on the MIM. Actually, different parameter ϖ can lead to different properties and performances for this information measure. In particular, to measure a distribution $P\left(X\right)=\{p\left(x_{1}\right),p\left(x_{2}\right),\ldots ,p\left(x_{n}\right)\}$, there are three kinds of work regions of MIM which can be classified by the parameters, whose details are discussed as follows.

(i) If the parameter satisfies $0\leq \varpi \leq 2/\max\{p\left(x_{i}\right)\}$, the convexity of MIM is similar to Shannon entropy and Renyi entropy. Actually, these three information measures all have maximum value properties and allocate weights for probability elements of the distribution P(X). It is notable that the MIM in this work region focuses on the typical sets rather than atypical sets, which implies that the uniform distribution reaches the maximum value. In brief, the MIM in this work region can be regarded as the same class of message measure as Shannon entropy and Renyi entropy to deal with the problems of information theory.
(ii) If we have $\varpi >2/\max\{p\left(x_{i}\right)\}$, the small probability elements will be the dominant factor for MIM to measure a distribution. That is, the small probability events can be highlighted more in this work region of MIM than those in the first one. Moreover, in this work region, MIM can pay more attention to atypical sets, which can be viewed as a magnifier for rare events. In fact, this property corresponds to some common scenarios where anomalies catch more eyes such as anomalous detection and alarm. In this case, some problems (including communication and probabilistic events processing) can be rehandled from the perspective of rare events importance. Particularly, the compression encoding and maximum entropy rate transmission are proposed based on the non-parametric MIM (namely NMIM) [24]; in addition, the distribution goodness-of-fit approach is also presented by use of the differential MIM (namely DMIM) [29].
(iii) If the MIM has the parameter ϖ<0, the large probability elements will be the main part contributing to the value of this information measure. In other words, the normal events attract more attention in this work region of MIM than rare events. In practice, this can be used in many applications where regular events are popular such as filter systems and data cleaning.

As a matter of fact, by selecting the parameter ϖ properly, we can exploit the MIM to solve several problems in different scenarios. The importance coefficient facilitates more flexibility of MIM in applications beyond Shannon entropy and Renyi entropy.

To focus on a concrete object, in this paper, we mainly investigate the first work region of MIM (namely $0\leq \varpi \leq 2/\max\{p\left(x_{i}\right)\}$) and intend to dig out some novelties related to this metric for information processing.

### 1.3. Similarities and Differences between Shannon Entropy and MIM

In fact, when the parameter ϖ satisfies $0\leq \varpi \leq 2/\max\{p\left(x_{i}\right)\}$, MIM is similar to Shannon entropy in regard to the expression and properties. The exponential operator of MIM is a substitute for the logarithm operator of Shannon entropy. As a kind of tool based on probability distributions, the MIM with parameter $0\leq \varpi \leq 2/\max\{p\left(x_{i}\right)\}$ has the same concavity and monotonicity as Shannon entropy, which can characterize the information otherness for different variables.

By resorting to the exponential operator of MIM, the weights for small probability elements are amplified more in some degree than those for large probability ones, which is considered as message importance allocation based on the self-scoring values. In this regard, the MIM may add fresh factors to the information processing, which takes into account the effects of probabilistic events’ importance from an objective viewpoint.

In the conventional Shannon information theory, data transmission and compression both can be viewed as the information transfer process from the variable *X* to *Y*. The capacity of information transmission is achieved by maximizing the mutual information between the *X* and *Y*. Actually, there exists distortion for probabilistic events during an information transfer process, which denotes the difference between the source and its corresponding reconstruction. Due to this fact, it is possible to compress data based on the allowable information loss in a certain extent [30,31,32]. In Shannon information theory, rate-distortion theory is investigated for lossy data compression, whose essence is mutual information minimization under the constraint of a certain distortion. However, in some cases involved with distortion, small probability events containing more message importance require higher reliability than those with large probability. In this sense, another aspect of information distortion may be essential, in which message importance is considered as a reasonable metric. Particularly, information transfer process is characterized by the MIM (rather than the entropy) with controlling the distortion, which can be viewed as a new kind of information compression, compared to the conventional scheme compressing redundancy to save resources. In fact, some information measures with respect to message importance have been investigated to extend the range of Shannon information theory [33,34,35,36,37]. In this regard, it is worthwhile exploring the information processing in the sense of MIM. Furthermore, it is also promising to investigate the Shannon mutual information constrained by the MIM in an information transfer process which may become a novel system invariant.

In addition, similar to Shannon conditional entropy, a conditional message importance measure for two distributions is proposed to process conditional probability.

**Definition** **2.** *For the two discrete probability P(X) = $\{p\left(x_{1}\right)$, $p(x_{2})$, …, $p(x_{n})\}$ and P(Y) = $\{p\left(y_{1}\right)$, $p(y_{2})$, …, $p(y_{n})\}$, the conditional message importance measure (CMIM) is given by*

(4) $$\begin{array}{cc} L(\varpi ,X|Y) & =\sum_{y_{j}}p\left(y_{j}\right)\sum_{x_{i}}p\left(x_{i}|y_{j}\right)e^{\varpi \{1-p\left(x_{i}|y_{j}\right)\}}, \end{array}$$

*where $p(x_{i}|y_{j})$ denotes the conditional probability between $y_{j}$ and $x_{i}$. The component $p\left(x_{i}|y_{j}\right)e^{\varpi \{1-p\left(x_{i}|y_{j}\right)\}}$ is similar to self-scoring value. Therefore, the CMIM can be considered as a system invariant which indicates the average total self-scoring value for an information transfer process.*

Actually, the MIM is a metric with different mathematical and physical meaning from Shannon entropy and Renyi entropy, which provides its own perspective to process probabilistic events. However, due to the similarity between the MIM and Shannon entropy, they may have analogous performance in some aspects. To this end, the information processing based on the MIM is discussed in this paper.

### 1.4. Motivation and Contributions

**Figure 1:**
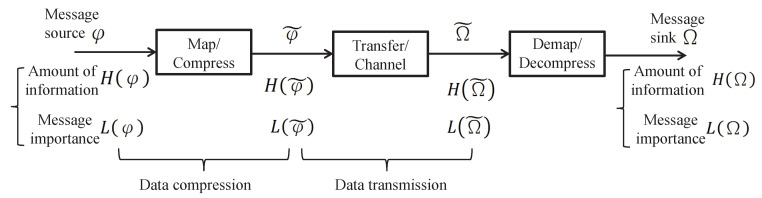
Information processing system model.

The purpose of this paper is to characterize the probabilistic events processing including compression and transmission by means of MIM. Particularly, in terms of the information processing system model shown in Figure 1, the message source φ (regarded as a random variable whose support set corresponds to the set of events’ types) can be measured by the amount of information H(·) and the message importance L(·) according to the probability distribution. Then, the information transfer process whose details are presented in Section 2 can be characterized based on these two metrics. Different from the mathematically probabilistic characterization of traditional telecommunication system, this paper mainly discusses the information processing from the perspectives of message importance. In this regard, the information importance harvest in a transmission is characterized by the proposed message importance loss capacity. Moreover, the upper bound of information compression based on the MIM is described by the message importance distortion function. In addition, we also investigate the trade-off between bitrate transmission and message importance loss to bring some inspiration to the conventional information theory.

### 1.5. Organization

The rest of this paper is discussed as follows. In Section 2, a system model involved with message importance is constructed to help analyze the data compression and transmission in big data. In Section 3, we propose a kind of message transfer capacity to investigate the message importance loss in the transmission. In Section 4, message importance distortion function is introduced and its properties are also presented to give some details. In Section 5, we discuss the bitrate transmission constrained by message importance to widen the horizon for the Shannon theory. In Section 6, some numerical results are presented to validate propositions and the analysis in theory. Finally, we conclude this paper in Section 7. Additionally, the fundamental notations in this paper are summarized in Table 1.

## 2. System Model with Message Importance

Considering an information processing system model shown in Figure 1, the information transfer process is discussed as follows. At first, a message source φ follows a distribution $P_{\varphi }=\left\{p\left(\varphi_{1}\right),p\left(\varphi_{2}\right),...,p\left(\varphi_{n}\right)\right\}$ whose support set is $\left\{\varphi_{1},\varphi_{2},...,\varphi_{n}\right\}$ corresponding to the events types. Then, the message φ is encoded or compressed into the variable $\tilde{\varphi }$ following the distribution $P_{\tilde{\varphi }}=\left\{p\left({\tilde{\varphi }}_{1}\right),p\left({\tilde{\varphi }}_{2}\right),...,p\left({\tilde{\varphi }}_{n}\right)\right\}$ whose alphabet is $\left\{\varphi_{1},\varphi_{2},...,\varphi_{n}\right\}$. After the information transfer process denoted by matrix $p({\tilde{\Omega }}_{j}|{\tilde{\varphi }}_{i})$, the received message $\tilde{\Omega }$ originating from $\tilde{\varphi }$ is observed as a random variable, where the distribution of $\tilde{\Omega }$ is $P_{\tilde{\Omega }}=\left\{p\left({\tilde{\Omega }}_{1}\right),p\left({\tilde{\Omega }}_{2}\right),...,p\left({\tilde{\Omega }}_{n}\right)\right\}$ whose alphabet is $\left\{{\tilde{\Omega }}_{1},{\tilde{\Omega }}_{2},...,{\tilde{\Omega }}_{n}\right\}$. Finally, the receiver recovers the original message φ by decoding $\Omega =g(\tilde{\Omega })$ where g(·) denotes the decoding function and Ω is the recovered message with the alphabet $\left\{\Omega_{1},\Omega_{2},...,\Omega_{n}\right\}$.

From the viewpoint of generalized information theory, a two-layer framework is considered to understand this model, where the first layer is based on the amount of information characterized by Shannon entropy denoted by H(·), while the second layer reposes on message importance measure of events denoted by L(·). Due to the fact that the former is discussed pretty entirely, we mainly investigate the latter in the paper.

Considering the source-channel separation theorem [38], the above information processing model consists of two problems, namely data compression and data transmission. On one hand, the *data compression* of the system can be achieved by using classical source coding strategies to reduce more redundancy, in which the information loss is described by $H\left(\varphi \right)-H\left(\varphi |\tilde{\varphi }\right)$ under the information transfer matrix $p(\tilde{\varphi }|\varphi )$. Similarly, from the perspective of message importance, the data can be further compressed by discarding worthless messages, where the message importance loss can be characterized by $L\left(\varphi \right)-L\left(\varphi |\tilde{\varphi }\right)$. On the other hand, the *data transmission* is discussed to obtain the upper bound of the mutual information $H\left(\tilde{\varphi }\right)-H\left(\tilde{\varphi }|\tilde{\Omega }\right)$, namely the information capacity. In a similar way, $L\left(\tilde{\varphi }\right)-L\left(\tilde{\varphi }|\tilde{\Omega }\right)$ means the income of message importance in the transmission.

In essence, it is apparent that the data compression and transmission are both considered as an information transfer processes {X,p(y|x),Y}, and they can be characterized by the difference between {X} and {X|Y}. In order to facilitate the analysis of the above model, the message importance loss is introduced as follows.

**Definition** **3.** *For two discrete probability P(X) = $\{p\left(x_{1}\right)$, $p(x_{2})$, …, $p(x_{n})\}$ and P(Y) = $\{p\left(y_{1}\right)$, $p(y_{2})$, …, $p(y_{n})\}$, the message importance loss based on MIM and CMIM is given by*

(5) $$\begin{array}{cc} \Phi_{\varpi }\left(X||Y\right) & =L(\varpi ,X)-L(\varpi ,X|Y), \end{array}$$

*where L(ϖ,X) and L(ϖ,X|Y) are given by the Definitions 1 and 2.*

In fact, according to the intrinsic relationship between L(ϖ,X) and L(ϖ,X|Y), it is readily seen that

(6) $$\begin{array}{c} \Phi_{\varpi }\left(X||Y\right)\geq 0, \end{array}$$

where $0<\varpi \leq 2\leq 2/\max\{p(x_{i}|y_{j})\}$.

**Proof.**  Considering a function $f\left(x\right)=xe^{\varpi (1-x)}$ (0≤x≤1 and 0<ϖ), it is easy to have $\frac{\partial^{2}f\left(x\right)}{\partial x}=-\varpi e^{\varpi (1-x)}\left(2-\varpi x\right)$, which implies if ϖ≤2≤2/x, the function f(x) is concave.

In the light of Jensen’s inequality, if $0<\varpi \leq 2\leq 2/\max\{p(x_{i}|y_{j})\}$ is satisfied, it is not difficult to see

(7) $$\begin{array}{cc} L(\varpi ,X) & =\sum_{x_{i}}p\left(x_{i}\right)e^{\varpi (1-p\left(x_{i}\right))}\\ & =\sum_{x_{i}}\left\{\sum_{y_{j}}p\left(y_{j}\right)p\left(x_{i}|y_{j}\right)\right\}e^{\varpi (1-\left\{\sum_{y_{j}}p\left(y_{j}\right)p\left(x_{i}|y_{j}\right)\right\})}\\ & \geq \sum_{y_{j}}p\left(y_{j}\right)\sum_{x_{i}}\left\{p\left(x_{i}|y_{j}\right)e^{\varpi (1-p\left(x_{i}|y_{j}\right))}\right\}=L\left(\varpi ,X|Y\right). \end{array}$$

□

## 3. Message Importance Loss in Transmission

In this section, we will introduce the CMIM to characterize the information transfer processing. To do so, we define a kind of message transfer capacity measured by the CMIM as follows.

**Definition** **4.** *Assume that there exists an information transfer process as*

(8) $$\begin{array}{c} \{X,p(y|x),Y\}, \end{array}$$

*where the p(y|x) denotes a probability distribution matrix describing the information transfer from the variable X to Y. We define the message importance loss capacity (MILC) as*

(9) $$\begin{array}{cc} C & =\max_{p(x)}\{\Phi_{\varpi }\left(X||Y)\right\}\\ & =\max_{p(x)}\left\{L\left(\varpi ,X\right)-L\left(\varpi ,X|Y\right)\right\}, \end{array}$$

*where $L\left(\varpi ,X\right)=\sum_{x_{i}}p\left(x_{i}\right)e^{\varpi \{1-p\left(x_{i}\right)\}}$, $p\left(y_{j}\right)=\sum_{x_{i}}p\left(x_{i}\right)p\left(y_{j}|x_{i}\right)$, $p\left(x_{i}|y_{j}\right)=\frac{p\left(x_{i}\right)p\left(y_{j}|x_{i}\right)}{p(y_{j})}$, L(ϖ,X|Y) is defined by Equation (4), and $\varpi <2\leq 2/\max\left\{p\left(x_{i}\right)\right\}$.*

In order to have an insight into the applications of MILC, some specific information transfer scenarios are discussed as follows.

### 3.1. Binary Symmetric Matrix

Consider the binary symmetric information transfer matrix, where the original variables are complemented with the transfer probability which can be seen in the following proposition.

**Proposition** **1.** *Assume that there exists an information transfer process {X,p(y|x),Y}, where the information transfer matrix is*

(10) $$\begin{array}{c} p\left(y|x\right)=\left[\begin{array}{cc} 1-\beta_{s} & \beta_{s}\\ \beta_{s} & 1-\beta_{s} \end{array}\right], \end{array}$$

*which indicates that X and Y both follow binary distributions. In that case, we have*

(11) $$\begin{array}{c} C\left(\varpi ,\beta_{s}\right)=e^{\frac{\varpi }{2}}-L\left(\varpi ,\beta_{s}\right), \end{array}$$

*where $L\left(\varpi ,\beta_{s}\right)=\beta_{s}e^{\varpi (1-\beta_{s})}+\left(1-\beta_{s}\right)e^{\varpi \beta_{s}}$ ($0\leq \beta_{s}\leq 1$) and $\varpi <2\leq 2/\max\left\{p\left(x_{i}\right)\right\}$.*

**Proof** **of** **Proposition** **1.** Assume that the distribution of variable *X* is a binary distribution (p,1−p). According to Equation (10) and Bayes’ theorem (namely, $p\left(x|y\right)=\frac{p(x)p(y|x)}{p(y)}$), it is not difficult to see that

(12) $$\begin{array}{c} p\left(x|y\right)=\left[\begin{array}{cc} \frac{p(1-\beta_{s})}{p\left(1-\beta_{s}\right)+\left(1-p\right)\beta_{s}} & \frac{\left(1-p\right)\beta_{s}}{p\left(1-\beta_{s}\right)+\left(1-p\right)\beta_{s}}\\ \frac{p\beta_{s}}{p\beta_{s}+\left(1-p\right)\left(1-\beta_{s}\right)} & \frac{\left(1-p\right)\left(1-\beta_{s}\right)}{p\beta_{s}+\left(1-p\right)\left(1-\beta_{s}\right)} \end{array}\right]. \end{array}$$

Furthermore, in accordance with Equations (4) and (9), we have

(13) $$\begin{array}{c} C(\varpi ,\beta_{s})\\ =\max_{p}\left\{C\left(p,\varpi ,\beta_{s}\right)\right\}\\ =\max_{p}\{L\left(\varpi ,p\right)-\{p\left(1-\beta_{s}\right)e^{\frac{\varpi \left(1-p\right)\beta_{s}}{p\left(1-\beta_{s}\right)+\left(1-p\right)\beta_{s}}}+\left(1-p\right)\beta_{s}e^{\frac{\varpi p(1-\beta_{s})}{p\left(1-\beta_{s}\right)+\left(1-p\right)\beta_{s}}}+p\beta_{s}e^{\frac{\varpi \left(1-p\right)\left(1-\beta_{s}\right)}{p\beta_{s}+\left(1-p\right)\left(1-\beta_{s}\right)}}\\ \;+\left(1-p\right)\left(1-\beta_{s}\right)e^{\frac{\varpi p\beta_{s}}{p\beta_{s}+\left(1-p\right)\left(1-\beta_{s}\right)}}\}\}, \end{array}$$

where $L\left(\varpi ,p\right)=pe^{\varpi (1-p)}+\left(1-p\right)e^{\varpi p}$ (0<p<1). Then, it is readily seen that

(14) $$\begin{array}{cc} \frac{\partial C(p,\varpi ,\beta_{s})}{\partial p}= & \left(1-\varpi p\right)e^{\varpi (1-p)}+\left[\left(1-p\right)\varpi -1\right]e^{\varpi p}\\ & -\{\left(1-\beta_{s}\right)\left\{1-\frac{\varpi p\left(1-\beta_{s}\right)\beta_{s}}{{\left[p\left(1-\beta_{s}\right)+\left(1-p\right)\beta_{s}\right]}^{2}}\right\}e^{\frac{\varpi (1-p)\beta }{p(1-\beta )+(1-p)\beta }}\\ & +\left(1-\beta_{s}\right)\left\{\frac{\varpi \left(1-p\right)\beta_{s}\left(1-\beta_{s}\right)}{{\left[p\beta_{s}+\left(1-p\right)\left(1-\beta_{s}\right)\right]}^{2}}-1\right\}e^{\frac{\varpi p\beta_{s}}{p\beta_{s}+\left(1-p\right)\left(1-\beta_{s}\right)}}\\ & +\beta_{s}\left\{\frac{\varpi \left(1-p\right)\beta_{s}\left(1-\beta_{s}\right)}{{\left[p\left(1-\beta_{s}\right)+\left(1-p\right)\beta_{s}\right]}^{2}}-1\right\}e^{\frac{\varpi p(1-\beta_{s})}{p\left(1-\beta_{s}\right)+\left(1-p\right)\beta_{s}}}\\ & +\beta_{s}\left\{1-\frac{\varpi p\left(1-\beta_{s}\right)\beta_{s}}{{\left[p\beta_{s}+\left(1-p\right)\left(1-\beta_{s}\right)\right]}^{2}}\right\}e^{\frac{\varpi \left(1-p\right)\left(1-\beta_{s}\right)}{p\beta_{s}+\left(1-p\right)\left(1-\beta_{s}\right)}}\}. \end{array}$$

In the light of the positivity for $\frac{\partial C(p,\beta_{s})}{\partial p}$ in {p|p∈(0,1/2)} and the negativity in {p|p∈(1/2,1)} (if $\beta_{s}\neq 1/2$), it is apparent that p=1/2 is the only solution for $\frac{\partial C(p,\beta_{s})}{\partial p}=0$. That is, if $\beta_{s}\neq 1/2$, the extreme value is indeed the maximum value of $C(p,\varpi ,\beta_{s})$ when p=1/2. Similarly, if $\beta_{s}=1/2$, the solution p=1/2 also results in the same conclusion. □

**Remark** **1.** *According to Proposition 1, on one hand, when $\beta_{s}=1/2$, that is, the information transfer process is just random, we will gain the lower bound of the MILC namely $C(\beta_{s})=0$. On the other hand, when $\beta_{s}=0$, namely there is a certain information transfer process, we will have the maximum MILC. As for the distribution selection for the variable X, the uniform distribution is preferred to gain the capacity.*

### 3.2. Binary Erasure Matrix

The binary erasure information transfer matrix is similar to the binary symmetric one; however, in the former, a part of information is lost rather than corrupted. The MILC of this kind of information transfer matrix is discussed as follows.

**Proposition** **2.** *Consider an information transfer process {X,p(y|x),Y}, in which the information transfer matrix is described as*

(15) $$\begin{array}{c} p\left(y|x\right)=\left[\begin{array}{ccc} 1-\beta_{e} & 0 & \beta_{e}\\ 0 & 1-\beta_{e} & \beta_{e} \end{array}\right], \end{array}$$

*which indicates that X follows the binary distribution and Y follows the 3-ary distribution. Then, we have*

(16) $$\begin{array}{cc} C(\varpi ,\beta_{e}) & =\left(1-\beta_{e}\right)\left\{e^{\frac{\varpi }{2}}-1\right\}, \end{array}$$

*where $0\leq \beta_{e}\leq 1$ and $0<\varpi <2\leq 2/\max\left\{p\left(x_{i}\right)\right\}$.*

**Proof** **of** **Proposition** **2.** Assume the distribution of variable *X* is (p,1−p). Furthermore, according to the binary erasure matrix and Bayes theorem, we have that the transmission matrix conditioned by the variable *Y* as follows:

(17) $$\begin{array}{c} p\left(x|y\right)=\left[\begin{array}{cc} 1 & 0\\ 0 & 1\\ p & 1-p \end{array}\right]. \end{array}$$

Then, it is not difficult to have

(18) $$\begin{array}{cc} L(\varpi ,X|Y) & =\beta_{e}pe^{\varpi (1-p)}+\beta_{e}\left(1-p\right)e^{\varpi p}+1-\beta_{e}. \end{array}$$

Furthermore, it is readily seen that

(19) $$\begin{array}{cc} C(p,\varpi ,\beta_{e}) & =\max_{p}\left\{L\left(\varpi ,p\right)-\left\{\beta_{e}pe^{\varpi (1-p)}+\beta_{e}\left(1-p\right)e^{\varpi p}+1-\beta_{e}\right\}\right\}\\ & =\left(1-\beta_{e}\right)\left\{\max_{p}\left\{L\left(\varpi ,p\right)\right\}-1\right\}, \end{array}$$

where $L\left(\varpi ,p\right)=pe^{\varpi (1-p)}+\left(1-p\right)e^{\varpi p}$. Moreover, we have the solution p=1/2 leads to $\frac{\partial L(\varpi ,p)}{\partial p}=0$ and the corresponding second derivative is

(20) $$\begin{array}{cc} \frac{\partial^{2}L\left(\varpi ,p\right)}{\partial p^{2}} & =e^{\varpi (1-p)}\left(\varpi p-2\right)\varpi +e^{\varpi p}\left[\left(1-p\right)\varpi -2\right]\varpi <0, \end{array}$$

which results from the condition $0<\varpi <2\leq 2/\max\left\{p\left(x_{i}\right)\right\}$.

Therefore, it is readily seen that, in the case p=1/2, the capacity $C(p,\varpi ,\beta_{e})$ reaches the maximum value. □

**Remark** **2.** *Proposition 2 indicates that, in the case $\beta_{e}=1$, the lower bound of the capacity is obtained, that is $C(\beta_{e})=0$. However, if a certain information transfer process is satisfied (namely $\beta_{e}=0$), we will have the maximum MILC. Similar to Proposition 1, the uniform distribution is selected to reach the capacity in practice.*

### 3.3. Strongly Symmetric Backward Matrix

As for a strongly symmetric backward matrix, it is viewed as a special example of information transmission. The discussion for the message transfer capacity in this case is similar to that in the symmetric matrix, whose details are given as follows.

**Proposition** **3.** *For an information transmission from the source X to the sink Y, assume that there exists a strongly symmetric backward matrix as follows:*

(21) $$\begin{array}{c} p\left(x|y\right)=\left[\begin{array}{cccc} 1-\beta_{k} & \frac{\beta_{k}}{K-1} & ... & \frac{\beta_{k}}{K-1}\\ \frac{\beta_{k}}{K-1} & 1-\beta_{k} & ... & \frac{\beta_{k}}{K-1}\\ ... & ... & ... & ...\\ \frac{\beta_{k}}{K-1} & ... & \frac{\beta_{k}}{K-1} & 1-\beta_{k} \end{array}\right], \end{array}$$

*which indicates that X and Y both obey K-ary distribution. We have*

(22) $$\begin{array}{c} C\left(\varpi ,\beta_{k}\right)=e^{\frac{\varpi (K-1)}{K}}-\left\{\left(1-\beta_{k}\right)e^{\varpi \beta_{k}}+\beta_{k}e^{\varpi (1-\frac{\beta_{k}}{K-1})}\right\}, \end{array}$$

*where $0\leq \beta_{k}\leq 1$, K≥2 and $0<\varpi <2\leq 2/\max\left\{p\left(x_{i}\right)\right\}$.*

**Proof** **of** **Proposition** **3.** For given *K*-ary variables *X* and *Y* whose distribution are $\left\{p\left(x_{1}\right),p\left(x_{2}\right),...,p\left(x_{K}\right)\right\}$ and $\left\{p\left(y_{1}\right),p\left(y_{2}\right),...,p\left(y_{K}\right)\right\}$ respectively, we can use the strongly symmetric backward matrix to obtain the relationship between the two variables as follows:

(23) $$\begin{array}{cc} p(x_{i}) & =\left(1-\beta_{k}\right)p\left(y_{i}\right)+\frac{\beta_{k}}{K-1}\left[1-p\left(y_{i}\right)\right]\;\left(i=1,2,...,K\right), \end{array}$$

which implies $p(x_{i})$ is a one-to-one onto function for $p(y_{i})$.

In accordance with Definition 2, it is easy to see that

(24) $$\begin{array}{cc} L(\varpi ,X|Y) & =\sum_{x_{i}}\sum_{y_{j}}p\left(y_{j}\right)p\left(x_{i}|y_{j}\right)e^{\varpi (1-p\left(x_{i}|y_{j}\right))}\\ & =\sum_{y_{j}}p\left(y_{j}\right)\left\{\left(1-\beta_{k}\right)e^{\varpi \beta_{k}}+\beta_{k}e^{\varpi (1-\frac{\beta_{k}}{K-1})}\right\}\\ & =\left(1-\beta_{k}\right)e^{\varpi \beta_{k}}+\beta_{k}e^{\varpi (1-\frac{\beta_{k}}{K-1})}. \end{array}$$

Moreover, by virtue of the definition of MILC in Equation (9), it is readily seen that

(25) $$\begin{array}{cc} C(\varpi ,\beta_{k}) & =\max_{p(x)}\left\{L\left(\varpi ,X\right)\right\}-\left[\;\left(1-\beta_{k}\right)e^{\varpi \beta_{k}}+\beta_{k}e^{\varpi (1-\frac{\beta_{k}}{K-1})}\right], \end{array}$$

where $L\left(\varpi ,X\right)=\sum_{x_{i}}p\left(x_{i}\right)e^{\varpi \{1-p\left(x_{i}\right)\}}$.

Then, by using Lagrange multiplier method, we have

(26) $$\begin{array}{cc} G(p\left(x_{i}\right),\lambda_{0}) & =\sum_{x_{i}}p\left(x_{i}\right)e^{\varpi (1-p\left(x_{i}\right))}+\lambda_{0}\left[\sum_{x_{i}}p\left(x_{i}\right)-1\right]. \end{array}$$

By setting $\frac{\partial G(p\left(x_{i}\right),\lambda_{0})}{\partial p(x_{i})}=0$ and $\frac{\partial G(p\left(x_{i}\right),\lambda_{0})}{\partial \lambda_{0}}=0$, it can be readily verified that the extreme value of $\sum_{y_{j}}p\left(y_{j}\right)e^{\varpi (1-p\left(y_{j}\right))}$ is achieved by the uniform distribution as a solution, that is $p\left(x_{1}\right)=p\left(x_{2}\right)=...=p\left(x_{K}\right)=1/K$. In the case that $0<\varpi <2\leq 2/\max\left\{p\left(x_{i}\right)\right\}$, we have $\frac{\partial^{2}G\left(p\left(x_{i}\right),\lambda_{0}\right)}{\partial p^{2}\left(x_{i}\right)}<0$ with respect to $p\left(x_{i}\right)\in \left[0,1\right]$, which implies that the extreme value of $\sum_{x_{i}}p\left(x_{i}\right)e^{\varpi (1-p\left(x_{i}\right))}$ is the maximum value.

In addition, according to the Equation (23), the uniform distribution of variable *X* is resulted from the uniform distribution for variable *Y*.

Therefore, by substituting the uniform distribution for p(x) into Equation (25), we will obtain the capacity $C(\varpi ,\beta_{k})$. □

Furthermore, in light of Equation (22), we have

(27) $$\begin{array}{cc} \frac{\partial C(\varpi ,\beta_{k})}{\partial \beta_{k}} & =\left\{1-\varpi \left(1-\beta_{k}\right)\right\}e^{\varpi \beta_{k}}+\left\{\frac{\varpi \beta_{k}}{K-1}-1\right\}e^{\varpi (1-\frac{\beta_{k}}{K-1})}. \end{array}$$

By setting $\frac{\partial C(\varpi ,\beta_{k})}{\partial \beta_{k}}=0$, it is apparent that $C(\varpi ,\beta_{k})$ reaches the extreme value in the case that $\beta_{k}=\frac{K-1}{K}$. Additionally, when the parameter ϖ satisfies $0<\varpi <2\leq 2/\max\left\{p\left(x_{i}\right)\right\}$, we also have the second derivative of the $C(\varpi ,\beta_{k})$ as follows:

(28) $$\begin{array}{cc} \frac{\partial^{2}C\left(\varpi ,\beta_{k}\right)}{\partial \beta_{k}^{2}} & =\varpi \left[2-\left(1-\beta_{k}\right)\varpi \right]e^{\varpi \beta_{k}}+\frac{\varpi }{K-1}\left\{2-\frac{\varpi \beta_{k}}{K-1}\right\}e^{\varpi (1-\frac{\beta_{k}}{K-1})}>0, \end{array}$$

which indicates that the convex $C(\varpi ,\beta_{k})$ reaches the minimum value 0 in the case $\beta_{k}=\frac{K-1}{K}$.

**Remark** **3.** *According to Proposition 3, when $\beta_{k}=\frac{K-1}{K}$, namely, the channel is just random, we gain the lower bound of the capacity namely $C(\varpi ,\beta_{k})=0$. On the contrary, when $\beta_{k}=0$ (that is, there is a certain channel), we will have the maximum capacity.*

## 4. Distortion of Message Importance Transfer

In this section, we will focus on the information transfer distortion, a common problem of information processing. In a real information system, there exists inevitable information distortion caused by noises or other disturbances, though the devices and hardware of telecommunication systems are updating and developing. Fortunately, there are still some bonuses from allowable distortion in some scenarios. For example, in conventional information theory, rate distortion is exploited to obtain source compression such as predictive encoding and hybrid encoding, which can save a lot of hardware resources and communication traffic [39].

Similar to the rate distortion theory for Shannon entropy [38], a kind of information distortion function based on MIM and CMIM is defined to characterize the effect of distortion on the message importance loss. In particular, there are some details of discussion as follows.

**Definition** **5.** *Assume that there exists an information transfer process {X,p(y|x),Y} from the variable X to Y, where the p(y|x) denotes a transfer matrix (distributions of X and Y are denoted by p(x) and p(y) respectively). For a given distortion function d(x,y) (d(x,y)≥0) and an allowable distortion D, the message importance distortion function is defined as*

(29) $$\begin{array}{cc} R_{\varpi }\left(D\right) & =\min_{p\left(y|x\right)\in B_{D}}\Phi_{\varpi }\left(X||Y\right)\\ & =\min_{p\left(y|x\right)\in B_{D}}\left\{L\left(\varpi ,X\right)-L\left(\varpi ,X|Y\right)\right\}, \end{array}$$

*in which $L\left(\varpi ,X\right)=\sum_{x_{i}}p\left(x_{i}\right)e^{\varpi \{1-p\left(x_{i}\right)\}}$, L(ϖ,X|Y) is defined by Equation (4), $0<\varpi \leq \frac{2\min_{j}\left\{p\left(y_{j}\right)\right\}}{\max_{i}\left\{p\left(x_{i}\right)\right\}}$ and $B_{D}=\left\{q\left(y|x\right):\bar{D}\leq D\right\}$ denotes the allowable information transfer matrix set where*

(30) $$\begin{array}{c} \bar{D}=\sum_{x_{i}}\sum_{y_{j}}p\left(x_{i}\right)p\left(y_{j}|x_{i}\right)d\left(x_{i},y_{j}\right), \end{array}$$

*which is the average distortion.*

In this model, the information source *X* is given and our goal is to select an adaptive p(y|x) to achieve the minimum allowable message importance loss under the distortion constraint. This provides a new theoretical guidance for information source compression from the perspective of message importance.

In contrast to the rate distortion of Shannon information theory, this new information distortion function just depends on the message importance loss rather than entropy loss to choose an appropriate information compression matrix. In practice, there are some similarities and differences between the rate distortion theory and the message importance distortion in terms of the source compression. On one hand, both two information distortion encodings can be regarded as special information transfer processes just with different optimization objectives. On the other hand, the new distortion theory tries to keep the rare events as high as possible, while the conventional rate distortion focuses on the amount of information itself. To some degree, by reducing more redundant common information, the new source compression strategy based on rare events (viewed as message importance) may save more computing and storage resources in big data.

### 4.1. Properties of Message Importance Distortion Function

In this subsection, we shall discuss some fundamental properties of rate distortion function based on message importance in details.

#### 4.1.1. Domain of Distortion

Here, we investigate the domain of allowable distortion, namely $\left[D_{\min},D_{\max}\right]$, and the corresponding message importance distortion function values as follows.

*(i)* The lower bound $D_{\min}$: Due to the fact $0\leq d(x_{i},y_{j})$, it is easy to obtain the non-negative average distortion, namely $0\leq \bar{D}$. Considering $\bar{D}\leq D$, we readily have the minimum allowable distortion, that is

(31) $$\begin{array}{c} D_{\min}=0, \end{array}$$

which implies the distortionless case, namely *Y* is the same as *X*.

In addition, when the lower bound $D_{\min}$ (namely the distortionless case) is satisfied, it is readily seen that

(32) $$\begin{array}{cc} L(\varpi ,X|Y) & =L\left(\varpi ,X|X\right)=\sum_{x_{i}}p\left(x_{i}\right)p\left(x_{i}|x_{i}\right)e^{\varpi \{1-p\left(x_{i}|x_{i}\right)\}}=1, \end{array}$$

and according to the Equation (29) the message importance distortion function is

(33) $$\begin{array}{cc} R_{\varpi }\left(D_{\min}\right) & =L(\varpi ,X)-L(\varpi ,X|X)=L(\varpi ,X)-1, \end{array}$$

where $L\left(\varpi ,X\right)=\sum_{x_{i}}p\left(x_{i}\right)e^{\varpi \{1-p\left(x_{i}\right)\}}$ and $0<\varpi \leq \frac{2\min_{j}\left\{p\left(y_{j}\right)\right\}}{\max_{i}\left\{p\left(x_{i}\right)\right\}}$.

*(ii)* The upper bound $D_{\max}$: When the allowable distortion satisfies $D\geq D_{\max}$, it is apparent that the variables *X* and *Y* are independent, that is, p(y|x)=p(y). Furthermore, it is not difficult to see that

(34) $$\begin{array}{cc} D_{\max} & =\min_{p(y)}\left\{\sum_{x_{i}}\sum_{y_{j}}p\left(x_{i}\right)p\left(y_{j}\right)d\left(x_{i},y_{j}\right)\right\}\\ & =\sum_{y_{j}}p\left(y_{j}\right)\min_{p(y)}\left\{\sum_{x_{i}}p\left(x_{i}\right)d\left(x_{i},y_{j}\right)\right\}\\ & \geq \min_{y_{j}}\left\{\sum_{x_{i}}p\left(x_{i}\right)d\left(x_{i},y_{j}\right)\right\}, \end{array}$$

which indicates that when the distribution of variable *Y* follows $p(y_{j})=1$ and $p(y_{l})=0$ (l≠j), we have the upper bound

(35) $$\begin{array}{c} D_{\max}=\min_{y_{j}}\left\{\sum_{x_{i}}p\left(x_{i}\right)d\left(x_{i},y_{j}\right)\right\}. \end{array}$$

Additionally, on account of the independent *X* and *Y*, namely p(x|y)=p(x), it is readily seen that

(36) $$\begin{array}{cc} R_{\varpi }\left(D_{\max}\right) & =L\left(\varpi ,X\right)-\sum_{y_{j}}p\left(y_{j}\right)L\left(\varpi ,X\right)=0. \end{array}$$

#### 4.1.2. The Convexity Property

For two allowable distortions $D_{a}$ and $D_{b}$, whose optimal allowable information transfer matrixes are $p_{a}\left(y|x\right)$ and $p_{b}\left(y|x\right)$ respectively, we have

(37) $$\begin{array}{c} R_{\varpi }\left(\delta D_{a}+\left(1-\delta \right)D_{b}\right)\leq \delta R_{\varpi }\left(D_{a}\right)+\left(1-\delta \right)R_{\varpi }\left(D_{b}\right), \end{array}$$

where 0≤δ≤1 and $0<\varpi \leq \frac{2\min_{j}\left\{p\left(y_{j}\right)\right\}}{\max_{i}\left\{p\left(x_{i}\right)\right\}}$.

**Proof.**  Refer to the Appendix A. □

#### 4.1.3. The Monotonically Decreasing Property

For two given allowable distortions $D_{a}$ and $D_{b}$, if $0\leq D_{a}<D_{b}<D_{\max}$ is satisfied, we have $R_{\varpi }\left(D_{a}\right)\geq R_{\varpi }\left(D_{b}\right)$, where $0<\varpi \leq \frac{2\min_{j}\left\{p\left(y_{j}\right)\right\}}{\max_{i}\left\{p\left(x_{i}\right)\right\}}$.

**Proof.**  Considering that $0\leq D_{a}<D_{b}<D_{\max}$, we have $D_{b}=\gamma D_{a}+\left(1-\gamma \right)D_{\max}$ where $\gamma =\frac{D_{\max}-D_{b}}{D_{\max}-D_{a}}$. On account of the Equation (36) and the convexity property mentioned in Equation (37), it is not difficult to see that

(38) $$\begin{array}{cc} R_{\varpi }\left(D_{b}\right) & \leq \gamma R_{\varpi }\left(D_{a}\right)+\left(1-\gamma \right)R_{\varpi }\left(D_{\max}\right)=\gamma R_{\varpi }\left(D_{a}\right)<R_{\varpi }\left(D_{a}\right), \end{array}$$

where 0<γ<1. □

#### 4.1.4. The Equivalent Expression

For an information transfer process {X,p(y|x),Y}, if we have a given distortion function d(x,y), an allowable distortion *D* and a average distortion $\bar{D}$ defined in Equation (30), the message importance distortion function defined in Equation (29) can be rewritten as

(39) $$\begin{array}{c} R_{\varpi }\left(D\right)=\min_{\bar{D}=D}\left\{L\left(\varpi ,X\right)-L\left(\varpi ,X|Y\right)\right\}, \end{array}$$

where L(ϖ,X) and L(ϖ,X|Y) are defined by the Equations (1) and (4), as well as $0<\varpi \leq \frac{2\min_{j}\left\{p\left(y_{j}\right)\right\}}{\max_{i}\left\{p\left(x_{i}\right)\right\}}$.

**Proof.**  For a given allowable distortion *D*, if there exists an allowable distortion $D^{*}$ ($D_{\min}\leq D^{*}<D<D_{\max}$) and the corresponding optimal information transfer matrix $p^{*}\left(y|x\right)$ leads to $R_{\varpi }\left(D\right)$, we will have $R_{\varpi }\left(D\right)=R_{\varpi }\left(D^{*}\right),$ which contradicts the monotonically decreasing property. □

### 4.2. Analysis for Message Importance Distortion Function

In this subsection, we shall investigate the computation of message importance distortion function, which has a great impact on the probabilistic events analysis in practice. Actually, the definition of message importance distortion function in Equation (29) can be regarded as a special function, which is the minimization of the message importance loss with the symbol error less than or equal to the allowable distortion *D*. In particular, Definition 5 can also be expressed as the following optimization:

(40) $$\begin{array}{cc} P_{1}:\min_{p(y_{j}|x_{i})}\;\;\; & \left\{L(\varpi ,X)-L(\varpi ,X|Y)\right\}\\ s.t.\;\;\; & \sum_{x_{i}}\sum_{y_{j}}p\left(x_{i}\right)p\left(y_{j}|x_{i}\right)d\left(x_{i},y_{j}\right)\leq D,\\ & \sum_{y_{j}}p\left(y_{j}|x_{i}\right)=1,\\ & p(y_{j}|x_{i})\geq 0, \end{array}$$

where L(ϖ,X) and L(ϖ,X|Y) are MIM and CMIM defined in Equations (1) and (4), as well as $0<\varpi \leq \frac{2\min_{j}\left\{p\left(y_{j}\right)\right\}}{\max_{i}\left\{p\left(x_{i}\right)\right\}}$.

To take a computable optimization problem as an example, we consider Hamming distortion as the distortion function d(x,y), namely

(41) $$\begin{array}{c} d\left(x,y\right)=\left[\begin{array}{cccc} 0 & 1 & \ldots & 1\\ 1 & 0 & \ldots & 1\\ \ldots & \ldots & \ldots \\ 1 & 1 & \ldots & 0 \end{array}\right], \end{array}$$

which means $d(x_{i},y_{i})=0$ and $d(x_{i},y_{j})=1$ (i≠j). In order to reveal some intrinsic meanings of $R_{\varpi }\left(D\right)$, we investigate an information transfer of Bernoulli source as follows.

**Proposition** **4.** *For a Bernoulli(p) source denoted by a variable X and an information transfer process {X,p(y|x),Y} with Hamming distortion, the message importance distortion function is given by*

(42) $$\begin{array}{c} \begin{array}{c} R_{\varpi }\left(D\right)=\left\{pe^{\varpi (1-p)}+\left(1-p\right)e^{\varpi p}\right\}-\left\{De^{\varpi (1-D)}+\left(1-D\right)e^{\varpi D}\right\}, \end{array} \end{array}$$

*and the corresponding information transfer matrix is*

(43) $$\begin{array}{c} p\left(y|x\right)=\left[\begin{array}{cc} \frac{\left(1-D)(p-D\right)}{p(1-2D)} & \frac{(1-p-D)D}{p(1-2D)}\\ \frac{D(p-D)}{\left(1-p)(1-2D\right)} & \frac{\left(1-p-D)(1-D\right)}{\left(1-p)(1-2D\right)} \end{array}\right], \end{array}$$

*where $0<\varpi \leq \frac{2\min_{j}\left\{p\left(y_{j}\right)\right\}}{\max_{i}\left\{p\left(x_{i}\right)\right\}}$ and 0≤D≤min{p,1−p}.*

**Proof** **of** **Proposition** **4.** Refer to the Appendix B. □

## 5. Bitrate Transmission Constrained by Message Importance

We investigate the information capacity in the case of a limited message importance loss in this section. The objective is to achieve the maximum transmission bitrate under the constraint of a certain message importance loss ϵ. The maximum transmission bitrate is one of system invariants in a transmission process, which provides a upper bound of amount of information obtained by the receiver.

In an information transmission process, the information capacity is the mutual information between the encoded signal and the received signal with the dimension bit/symbol. In a real transmission, there always exists an allowable distortion between the sending sequence *X* and the received sequence *Y*, while the maximum allowable message importance loss is required to avoid too much distortion of important events. From this perspective, message importance loss is considered to be another constraint for the information transmission capacity beyond the information distortion. Therefore, this might play a crucial role in the design of transmission in information processing systems.

In particular, we characterize the maximizing mutual information constrained by a controlled message importance loss as follows:

(44) $$\begin{array}{cc} P_{2}:\;\;\max_{p(x)}\;\;\; & I(X||Y)\\ s.t.\;\;\; & L(\varpi ,X)-L(\varpi ,X|Y)\leq \epsilon ,\\ & \sum_{y_{j}}p\left(x_{i}\right)=1,\\ & p(x_{i})\geq 0, \end{array}$$

where $I\left(X||Y\right)=\sum_{x_{i},y_{j}}p\left(x_{i}\right)p\left(y_{j}|x_{i}\right)\log\frac{p\left(x_{i}\right)p\left(y_{j}|x_{i}\right)}{p(y_{j})}$, $p\left(y_{j}\right)=\sum_{x_{i}}p\left(x_{i}\right)p\left(y_{j}|x_{i}\right)$, L(ϖ,X) and L(ϖ,X|Y) are MIM and CMIM defined in Equations (1) and (4), as well as $0<\varpi \leq \frac{2\min_{j}\left\{p\left(y_{j}\right)\right\}}{\max_{i}\left\{p\left(x_{i}\right)\right\}}$.

Actually, the bitrate transmission with a message importance loss constraint has a special solution for a certain scenario. In order to give a specific example, we investigate the optimization problem in the Bernoulli(*p*) source with a symmetric or erasure transfer matrix as follows.

### 5.1. Binary Symmetric Matrix

**Proposition** **5.** *For a Bernoulli(p) source X whose distribution is {p,1−p} (0≤p≤1/2) and an information transfer process {X,p(y|x),Y} with transfer matrix*

(45) $$\begin{array}{c} p\left(y|x\right)=\left[\begin{array}{cc} 1-\beta_{s} & \beta_{s}\\ \beta_{s} & 1-\beta_{s} \end{array}\right], \end{array}$$

*we have the solution for $P_{2}$ defined in Equation (44) as follows:*

(46) $$\begin{array}{c} \max_{p(x)}I\left(X||Y\right)\\ =\left\{\begin{array}{c} 1-H\left(\beta_{s}\right),\;\;\left(\epsilon \geq C_{\beta_{s}}\right)\\ H\left(p_{s}\left(1-\beta_{s}\right)+\left(1-p_{s}\right)\beta_{s}\right)-H\left(\beta_{s}\right),\;\;\left(0<\epsilon \leq C_{\beta_{s}}\right), \end{array}\right. \end{array}$$

*where $p_{s}$ is the solution of L(ϖ,X)−L(ϖ,X|Y)=ϵ (L(ϖ,X) and L(ϖ,X|Y) mentioned in the optimization problem $P_{2}$), whose approximate value is*

(47) $$\begin{array}{c} p_{s}≐\frac{1-\sqrt{\Theta }}{2}, \end{array}$$

*in which the parameter *Θ* is given by*

(48) $$\begin{array}{cc} \Theta & =1-\frac{4\epsilon }{4\varpi +\varpi^{2}}-\frac{4\sqrt{{\left(1-2\beta_{s}\right)}^{2}\epsilon^{2}+2\left(4\varpi +\varpi^{2}\right)\beta_{s}\left(1-\beta_{s}\right)\epsilon }}{\left(4\varpi +\varpi^{2}\right)\left|1-2\beta_{s}\right|}, \end{array}$$

*and H(·) denotes the operator for Shannon entropy, that is H(p)=−[(1−p)log(1−p)+plogp], $C_{\beta_{s}}=e^{\frac{\varpi }{2}}-\left\{\beta_{s}e^{\varpi (1-\beta_{s})}+\left(1-\beta_{s}\right)e^{\varpi \beta_{s}}\right\}$ ($0\leq \beta_{s}\leq 1$) and $\varpi <2\leq 2/\max\left\{p\left(x_{i}\right)\right\}$.*

**Proof** **of** **Proposition** **5.** Considering the Bernoulli(*p*) source *X* following {p,1−p} and the binary symmetric matrix, it is not difficult to gain

(49) $$\begin{array}{cc} I(X||Y) & =H(Y)-H(Y|X)\\ & =-\left\{p\left(y_{0}\right)\log p\left(y_{0}\right)+p\left(y_{1}\right)\log p\left(y_{1}\right)\right\}-H\left(\beta_{s}\right), \end{array}$$

where $p\left(y_{0}\right)=p\left(1-\beta_{s}\right)+\left(1-p\right)\beta_{s}$, $p\left(y_{1}\right)=p\beta_{s}+\left(1-p\right)\left(1-\beta_{s}\right)$ and $H\left(\beta_{s}\right)=-\left[\left(1-\beta_{s}\right)\log\left(1-\beta_{s}\right)+\beta_{s}\log\beta_{s}\right]$.

Moreover, define the Lagrange function as $G_{s}\left(p\right)=I\left(X||Y\right)+\lambda_{s}\left(L\left(\varpi ,X\right)-L\left(\varpi ,X|Y\right)-\epsilon \right)$ where ϵ>0, 0≤p≤1/2 and $\lambda_{s}\geq 0$. It is not difficult to have the partial derivative of $G_{s}\left(p\right)$ as follows:

(50) $$\begin{array}{c} \frac{\partial G_{s}\left(p\right)}{\partial p}=\frac{\partial I(X||Y)}{\partial p}+\lambda_{s}\frac{\partial C(p,\varpi ,\beta_{s})}{\partial p}, \end{array}$$

where $\frac{\partial C(p,\varpi ,\beta_{s})}{\partial p}$ is given by the Equation (14) and

(51) $$\begin{array}{c} \frac{\partial I(X||Y)}{\partial p}=\left(1-2\beta_{s}\right)\log\left\{\frac{\left(2\beta_{s}-1\right)p+1-\beta_{s}}{\left(1-2\beta_{s}\right)p+\beta_{s}}\right\}. \end{array}$$

By virtue of the monotonic increasing function log(x) for x>0, it is easy to see the nonnegativity of $\frac{\partial I(X||Y)}{\partial p}$ is equal to $\left(1-2\beta_{s}\right)\left\{\left(2\beta_{s}-1\right)p+1-\beta_{s}-\left[\left(1-2\beta_{s}\right)p+\beta_{s}\right]\right\}=\left(1-2p\right){\left(1-2\beta_{s}\right)}^{2}\geq 0$ in the case 0≤p≤1/2. Moreover, due to the nonnegative $\frac{\partial C(p,\varpi ,\beta_{s})}{\partial p}$ in p∈[0,1/2] which is mentioned in the proof of Proposition 1, it is readily seen that $\frac{\partial G_{s}\left(p\right)}{\partial p}\geq 0$ is satisfied under the condition 0≤p≤1/2.

Thus, the optimal solution $p_{s}^{*}$ is the maximal available *p* (p∈[0,1/2]) as follows:

(52) $$\begin{array}{c} p_{s}^{*}=\left\{\begin{array}{c} \frac{1}{2},\;\;\mathrm{for}\;\epsilon \geq C_{\beta_{s}},\\ p_{s},\;\;\mathrm{for}\;0<\epsilon \leq C_{\beta_{s}}, \end{array}\right. \end{array}$$

where $p_{s}$ is the solution of L(ϖ,X)−L(ϖ,X|Y)=ϵ, and $C_{\beta_{s}}$ is the MILC mentioned in Equation (11).

By using Taylor series expansion, the equation L(ϖ,X)−L(ϖ,X|Y)=ϵ can be expressed approximately as follows:

(53) $$\begin{array}{c} \left(2\varpi +\frac{\varpi^{2}}{2}\right)\left\{\left(1-p\right)p-\frac{p\left(1-p\right)\beta_{s}\left(1-\beta_{s}\right)}{\left[\left(2\beta_{s}-1\right)p+1-\beta_{s}\right]\left[\left(1-2\beta_{s}\right)p+\beta_{s}\right]}\right\}=\epsilon , \end{array}$$

whose solution is the approximate $p_{s}$ as the Equation (47).

Therefore, by substituting the $p_{s}^{*}$ into Equation (49), we have Equation (46). □

**Remark** **4.** *Proposition 5 gives the maximum transmission bitrate under the constraint of message importance loss. Particularly, there are growth regions and smooth regions for the maximum transmission bitrate in the receiver with respect to message importance loss ϵ. When the message importance loss ϵ is constrained in a little range, the real bitrate is less than the Shannon information capacity, which is involved with the entropy of the symmetric matrix parameter $\beta_{s}$.*

### 5.2. Binary Erasure Matrix

**Proposition** **6.** *Assume that there is a Bernoulli(p) source X following distribution {p,1−p} (0≤p≤1/2) and an information transfer process {X,p(y|x),Y} with the binary erasure matrix*

(54) $$\begin{array}{c} p\left(y|x\right)=\left[\begin{array}{ccc} 1-\beta_{e} & 0 & \beta_{e}\\ 0 & 1-\beta_{e} & \beta_{e} \end{array}\right], \end{array}$$

*where $0\leq \beta_{e}\leq 1$. In this case, the solution for $P_{2}$ described in Equation (44) is*

(55) $$\begin{array}{c} \max_{p(x)}I\left(X||Y\right)\\ =\left\{\begin{array}{c} 1-\beta_{e},\;\;\left(\epsilon \geq C_{\beta_{e}}\right)\\ \left(1-\beta_{e}\right)H\left(p_{e}\right),\;\;\left(0<\epsilon \leq C_{\beta_{s}}\right), \end{array}\right. \end{array}$$

*where $p_{e}$ is the solution of $\left(1-\beta_{e}\right)\left\{pe^{\varpi (1-p)}+\left(1-p\right)e^{\varpi p}-1\right\}=\epsilon$, whose approximate value is*

(56) $$\begin{array}{c} p_{e}≐\frac{1-\sqrt{1-\frac{8\epsilon }{\left(1-\beta_{e}\right)\left(4\varpi +\varpi^{2}\right)}}}{2}, \end{array}$$

*and H(x)=−[(1−x)log(1−x)+xlogx], $C_{\beta_{e}}=\left(1-\beta_{e}\right)\left(e^{\frac{\varpi }{2}}-1\right)$ and $\varpi <2\leq 2/\max\left\{p\left(x_{i}\right)\right\}$.*

**Proof** **of** **Proposition** **6.** In the binary erasure matrix, considering the Bernoulli(*p*) source *X* whose distribution is {p,1−p}, it is readily seen that

(57) $$\begin{array}{cc} I(X||Y) & =H(Y)-H(Y|X)\\ & =\left(1-\beta_{e}\right)H\left(p\right), \end{array}$$

where H(·) denotes the Shannon entropy operator, namely H(p)=−[(1−p)log(1−p)+plogp].

Moreover, according to the Definitions 1 and 2, it is easy to see that

(58) $$L\left(\varpi ,X\right)-L\left(\varpi ,X|Y\right)=\left(1-\beta_{e}\right)\left\{L\left(\varpi ,p\right)-1\right\},$$

where $L\left(\varpi ,p\right)=pe^{\varpi (1-p)}+\left(1-p\right)e^{\varpi p}$.

Similar to the proof of the Proposition 5 and considering the monotonically increasing H(p) and L(ϖ,p) in p∈[0,1/2], it is not difficult to see that the optimal solution $p_{e}^{*}$ is the maximal available *p* in the case $0\leq p\leq \frac{1}{2}$, which is given by

(59) $$\begin{array}{c} p_{e}^{*}=\left\{\begin{array}{c} \frac{1}{2},\;\;\mathrm{for}\;\epsilon \geq C_{\beta_{e}},\\ p_{e},\;\;\mathrm{for}\;0<\epsilon \leq C_{\beta_{e}}, \end{array}\right. \end{array}$$

where $p_{e}$ is the solution of $\left(1-\beta_{e}\right)\left\{L\left(\varpi ,p\right)-1\right\}=\epsilon$, and the upper bound $C_{\beta_{e}}$ is gained in Equation (16).

By resorting to Taylor series expansion, the approximate equation for $\left(1-\beta_{e}\right)\left\{L\left(\varpi ,p\right)-1\right\}=\epsilon$ is given by

(60) $$\begin{array}{c} \left(1-\beta_{e}\right)\left(2\varpi +\frac{\varpi^{2}}{2}\right)\left(1-p\right)p=\epsilon , \end{array}$$

from which the approximate solution $p_{e}$ in Equation (56) is obtained.

Therefore, Equation (55) is obtained by substituting the $p_{e}^{*}$ into the Equation (57). □

**Remark** **5.** *From Proposition 6, there are two regions for the maximum transmission bitrate with respect to message importance loss. The one depends on the message importance loss threshold ϵ. The other is just related to the erasure matrix parameter $\beta_{e}$.*

Note that single-letter models are discussed to show some theoretical results for information transfer under the constraint of massage importance loss, which may be used in some special potential applications such as maritime international signal or switch signal processing. As a matter of fact, in practice, it is preferred to operate multi-letters models which can be applied to more scenarios such as the multimedia communication, cooperative communications and multiple access, etc. As for these complicated cases which may be different from conventional Shannon information theory, we shall consider it in the near future.

## 6. Numerical Results

This section shall provide numerical results to validate the theoretical results in this paper.

### 6.1. The Message Importance Loss Capacity

**Figure 2:**
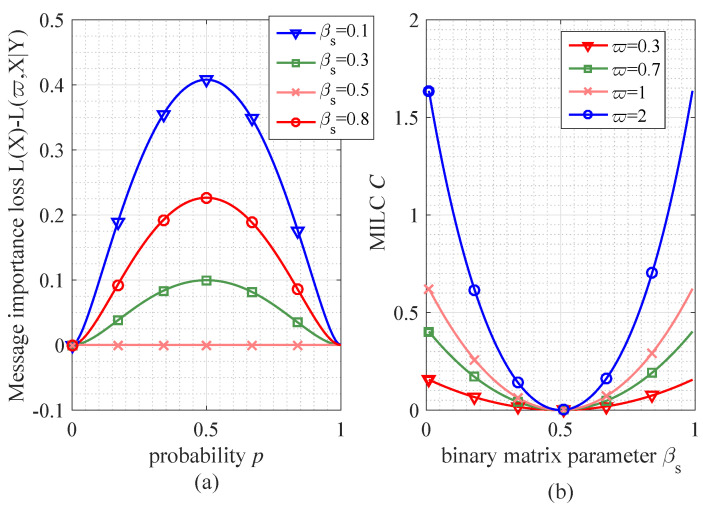
The performance of message importance loss and MILC (mentioned in Definition 4) in the Binary symmetric matrix. (**a**) the performance of message importance loss (with ϖ=1) versus probability *p* in the cases of different symmetric matrix parameter ($\beta_{s}=0.1,0.3,0.5,0.8$); (**b**) the performance of MILC versus matrix parameter $\beta_{s}$ in the cases of different parameter ϖ.

First of all, we give some numerical simulation with respect to the MILC in different information transmission cases. In Figure 2, it is apparent to see that if the Bernoulli source follows the uniform distribution, namely p=0.5, the message importance loss will reach the maximum in the cases of different matrix parameter $\beta_{s}$. That is, the numerical results of MILC are obtained as {0.4081,0.0997,0,0.2265} in the case of parameter $\beta_{s}=\left\{0.1,0.3,0.5,0.8\right\}$ and ϖ=1, which corresponds to Proposition 1. Moreover, we also know that if $\beta_{s}=0.5$, namely the random transfer matrix is satisfied, the MILC reaches the lower bound that is C=0. In contrast, if the parameter $\beta_{s}$ satisfies $\beta_{s}=0$, the upper bound of MILC will be gained such as {0.1618,0.4191,0.6487,1.7183} in the case ϖ={0.3,0.7,1.0,2.0}.

**Figure 3:**
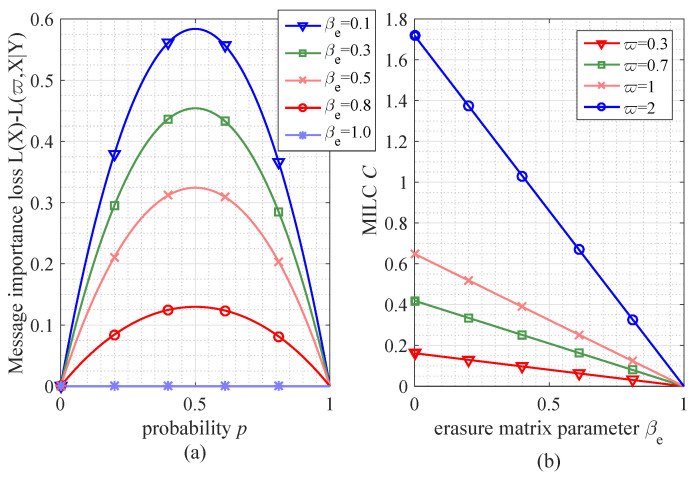
The performance of message importance loss and MILC in the Binary erasure matrix. (**a**) the performance of message importance loss (with ϖ=1) versus probability *p* in the cases of different matrix parameter ($\beta_{e}=0.1,0.3,0.5,0.8$); (**b**) the performance of MILC versus erasure matrix parameter $\beta_{e}$ in the cases of different parameter ϖ.

Figure 3 shows that, in the binary erasure matrix, the MILC is reached under the same condition as that in the binary symmetric matrix, namely p=0.5. For example the numerical results of MILC with ϖ=1 are {0.5838,0.4541,0.3244,0.1297} in the cases $\beta_{e}=\left\{0.1,0.3,0.5,0.8\right\}$. However, if $\beta_{e}=1$, the lower bound of MILC (C=0) is obtained in the erasure transfer matrix, different from the symmetric case.

**Figure 4:**
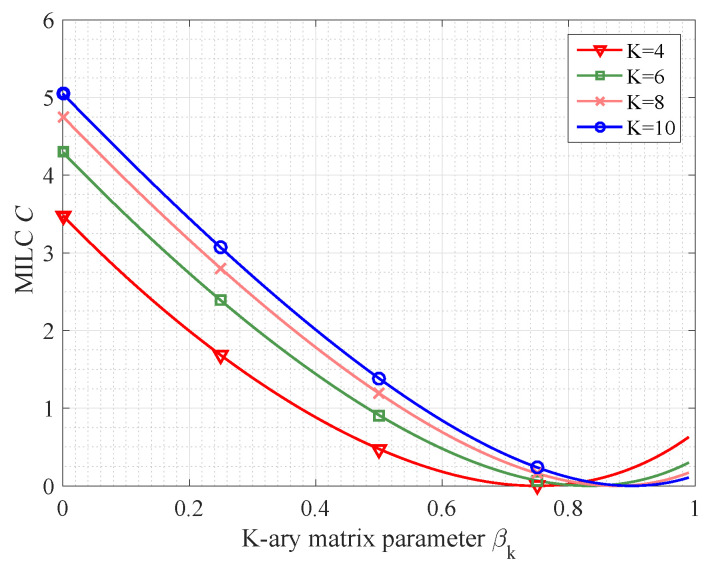
The performance of MILC in strongly symmetric matrix with K=4,6,8,10.

From Figure 4, it is not difficult to see that the certain transfer matrix (namely $\beta_{k}=0$) leads to upper bound of MILC. For example, when the number of source symbols satisfies K={4,6,8,10}, the numerical results of MILC with ϖ=2 are {3.4817,4.2945,4.7546,5.0496}. In addition, the lower bound of MILC is reached in the case that $\beta_{k}=1-\frac{1}{K}$.

### 6.2. Message Importance Distortion

**Figure 5:**
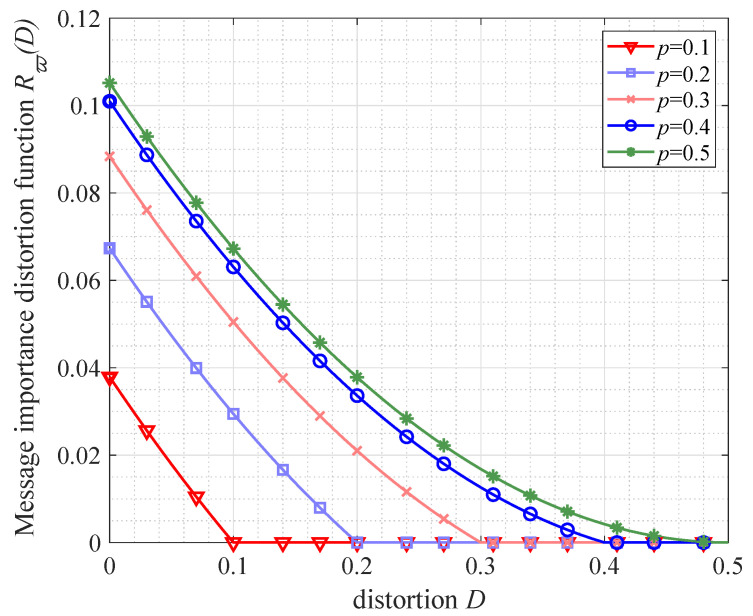
The performance of message importance distortion function $R_{\varpi }\left(D\right)$ in the case of Bernoulli(*p*) source (p=0.1,0.2,0.3,0.4).

We focus on the distortion of message importance transfer and give some simulations in this subsection. From Figure 5, it is illustrated that the message importance distortion function $R_{\varpi }\left(D\right)$ is monotonically non-increasing with respect to the distortion *D*, which can validate some properties mentioned in Section 4.1. Moreover, the maximum $R_{\varpi }\left(D\right)$ is obtained in the case D=0. Taking the Bernoulli(*p*) source as an example, the numerical results of $R_{\varpi }\left(D\right)$ with ϖ=0.2 are {0.0379,0.0674,0.0884,0.1010,0.1052} and the corresponding probability satisfies p={0.1,0.2,0.3,0.4,0.5}. Note that the turning point of $R_{\varpi }\left(D\right)$ is gained when the probability *p* equals to the distortion *D*, which conforms to Proposition 4.

### 6.3. Bitrate Transmission with Message Importance Loss

**Figure 6:**
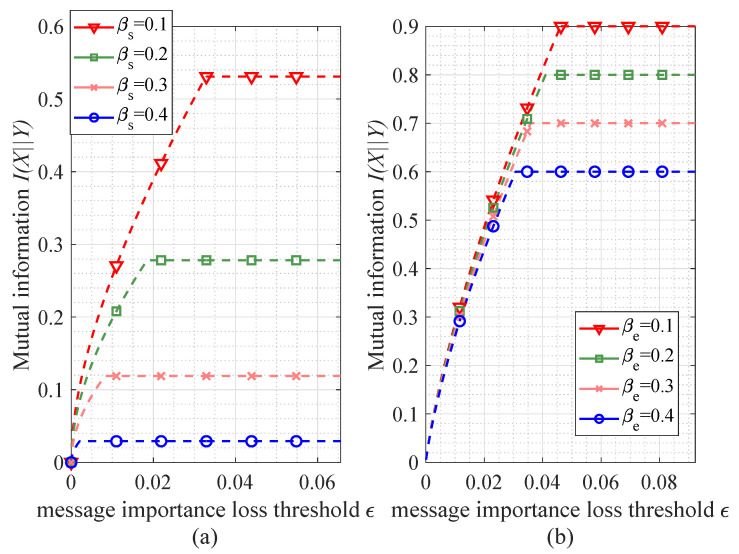
The performance of mutual information I(X||Y) constrained by the message importance loss ϵ (the parameter ϖ=0.1). (**a**) the performance of I(X||Y) versus ϵ in the binary symmetric matrix; (**b**) the performance of I(X||Y) versus ϵ in the erasure matrix.

Figure 6 shows the allowable maximum bitrate (characterized by mutual information) constrained by a message importance loss ϵ in a Bernoulli(*p*) source case. It is worth noting that there are two regions for the mutual information in the both transfer matrixes. In the first region, the mutual information is monotonically increasing with respect to the ϵ; however, in the second region, the mutual information is stable, namely the information transmission capacity is obtained. As for the numerical results, the turning points are obtained at ϵ={0.0328,0.0185,0.0082,0.0021} and the maximum mutual information values are {0.5310,0.2781,0.1187,0.0290} in the binary symmetric matrix with the corresponding parameter $\beta_{s}=\left\{0.1,0.2,0.3,0.4\right\}$, while the turning points of erasure matrix are at ϵ={0.0416,0.0410,0.0359,0.0308} in the case that $\beta_{e}=\left\{0.1,0.2,0.3,0.4\right\}$ with the maximum mutual information values {0.9,0.8,0.7,0.6}. Consequently, Propositions 5 and 6 are validated from the numerical results.

### 6.4. Experimental Simulations

In this subsection, we take the binary stochastic process (in which the random variable follows Bernoulli distribution) as an example to validate theoretical results. In particular, the Bernoulli(*p*) source *X* (whose distribution is denoted by P(X)={p,1−p} where 0<p<1) with the symmetric or erasure matrix (described by Equations (10) and (15)) is considered to reveal some properties of message importance loss capacity (in Section 3), message importance distortion function (in Section 4) as well as bitrate transmission constrained by message importance (in Section 5).

**Figure 7:**
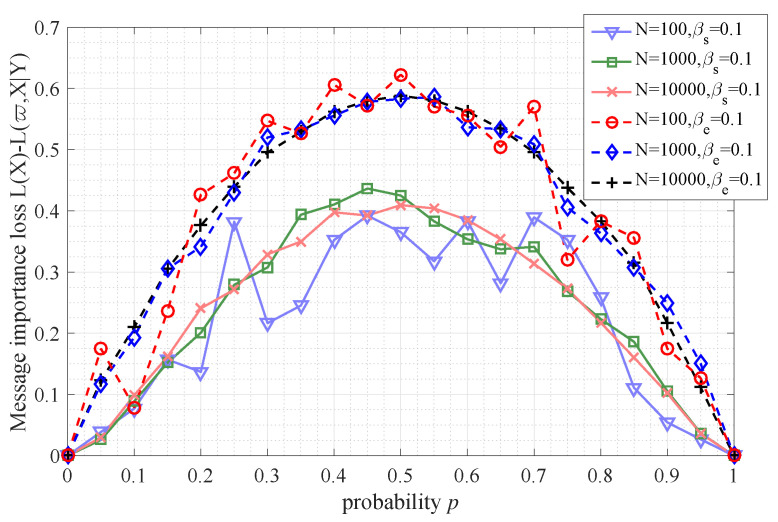
The message importance loss (with parameter ϖ=1) versus the probability *p* of Bernoulli(*p*) source with number of samples *N* (N={100,1000,10,000}). There are two different transfer matrices, namely the symmetric matrix with parameter $\beta_{s}=0.1$ and the erasure matrix with parameter $\beta_{e}=0.1$.

From Figure 7, it is seen that the uniform information source *X* (that is P(X)={1/2,1/2}) leads to the maximum message importance loss (namely MILC) in both cases of symmetric matrix and erasure matrix, which implies Propositions 1 and 2. Moreover, with the increase of number of samples, the performance of massage importance loss tends to smooth. In addition, the MILC in symmetric transfer matrix is larger than that in the erasure one when the matrix parameters $\beta_{s}$ and $\beta_{e}$ are the same.

**Figure 8:**
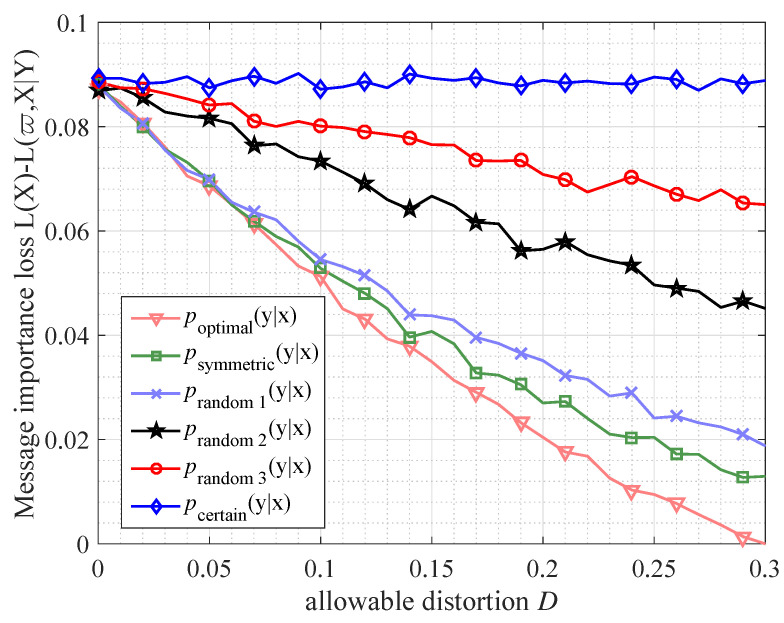
The message importance loss (with parameter ϖ=0.1) versus allowable distortion *D* (the corresponding distortion function is Hamming distortion) in the case of different transfer matrices. The information source *X* follows Bernoulli(*p*) distribution (where p=0.3, namely P(X)={0.3,0.7}) and the number of samples is n=10,000.

As for the distortion of message importance transfer, we investigate the message importance loss based on different transfer matrices, which is shown in Figure 8 where $p_{optimal}\left(y|x\right)$ is described as Equation (43), $p_{symmetric}\left(y|x\right)=\left[\begin{array}{cc} 1-D & D\\ D & 1-D \end{array}\right]$, $p_{random\;1}\left(y|x\right)=\left[\begin{array}{cc} 1-\frac{D}{10p} & \frac{D}{10p}\\ \frac{9D}{10(1-p)} & 1-\frac{9D}{10(1-p)} \end{array}\right]$, $p_{random\;2}\left(y|x\right)=\left[\begin{array}{cc} 1-\frac{D}{5p} & \frac{D}{5p}\\ \frac{D}{5(1-p)} & 1-\frac{D}{5(1-p)} \end{array}\right]$, $p_{random\;3}\left(y|x\right)=\left[\begin{array}{cc} 1-\frac{D}{10p} & \frac{D}{10p}\\ \frac{D}{10(1-p)} & 1-\frac{D}{10(1-p)} \end{array}\right]$, $p_{certain}\left(y|x\right)=\left[\begin{array}{cc} 1 & 0\\ 0 & 1 \end{array}\right]$, *D* is the allowable distortion and *p* is the probability element of Bernoulli(*p*) source. From Figure 8, it is illustrated that, when the $p_{optimal}\left(y|x\right)$ is selected as the transfer matrix, the massage importance loss reaches the minimum, which corresponds to Proposition 4. In addition, if the transfer matrix is not certain (existing distortion), message importance loss is decreasing with the increase of allowable distortion.

**Figure 9:**
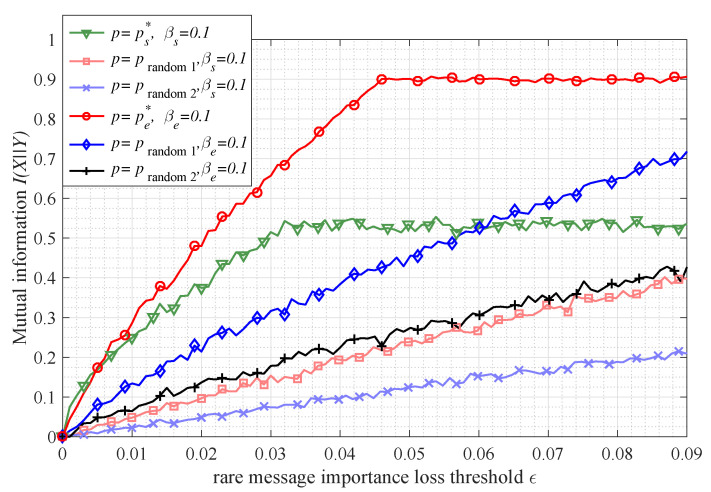
The mutual information I(X||Y) versus the rare message importance loss threshold ϵ (the parameter ϖ=0.1) in the case of Bernoulli(*p*) source *X* (that is P(X)={p,1−p} with different probability *p*). The number of samples observed from the source *X* is n=10,000, and transfer matrix is the symmetric matrix with parameter $\beta_{s}=0.1$ or the erasure matrix with parameter $\beta_{e}=0.1$.

Considering the transmission with a message importance loss constraint, Figure 9 shows that, when the $p_{s}^{*}$ (given by Equation (52)) and $p_{e}^{*}$ (given by Equation (59)) are selected as the probability elements for the Bernoulli(*p*) source in the symmetric matrix and erasure matrix respectively, the corresponding mutual information values are larger than those based on other probability (such as $p_{random\;1}=\left(1-\sqrt{1-8\epsilon }\right)/2$ and $p_{random\;2}=\left(1-\sqrt{1-4\epsilon }\right)/2$). In addition, it is not difficult to see that, when the parameter $\beta_{s}$ is equal to $\beta_{e}$, the mutual information (constrained by a message importance loss) in symmetric transfer matrix is larger than that in the erasure one.

## 7. Conclusions

In this paper, we investigated the information processing from the perspective of an information measure i.e., MIM. Actually, with the help of parameter ϖ, the MIM has more flexibility and can be used widely. Here, we just focused on the MIM with $0\leq \varpi \leq 2/\max\{p\left(x_{i}\right)\}$ which not only has properties of self-scoring values for probabilistic events but also has similarities with Shannon entropy in information compression and transmission. In particular, based on a system model with message importance processing, a message importance loss was presented. This measure can characterize the information distinction before and after a message transfer process. Furthermore, we have proposed the message importance loss capacity which can provide an upper bound for the message importance harvest in the information transmission. Moreover, the message importance distortion function, which is to select an information transfer matrix to minimize the message importance loss, was discussed to characterize the performance of information lossy compression from the viewpoint of message importance of events. In addition, we exploited the message importance loss to constrain the bitrate transmission so that the combined factors of message importance and amount of information are considered to guide an information transmission. To give the validation for theoretical analyses, some numerical results and experimental simulations were also presented in details. As the next step research, we are looking forward to exploiting real data to design some applicable strategies for information processing based on the MIM, as well as investigating the performance of multivariate systems in the sense of MIM. 

## Figures and Tables

**Table 1:** Notations.

Notation	Description
P(X) = $\{p\left(x_{1}\right)$, $p(x_{2})$, …, $p(x_{n})\}$	The discrete probability distribution with respect to the variable *X*
φ	The message source in the information processing system model
$\tilde{\varphi }$	The mapped or compressed message with respect to the φ
$\tilde{\Omega }$	The received message transferred from the $\tilde{\varphi }$
Ω	The recovered message with respect to the φ by the decoding process
ϖ	The importance coefficient
L(·)	The message importance measure (MIM) described as Definition 1
H(·)	The Shannon entropy, $H\left(X\right)=-\sum_{x_{i}}p\left(x_{i}\right)\log p(x_{i})$
	or H(p)=−plogp−(1−p)log(1−p),(0≤p≤1)
$H_{\alpha }\left(\cdot \right)$	The Renyi entropy with the parameter α $H_{\alpha }\left(X\right)=\frac{1}{1-\alpha }\log\sum_{x_{i}}{\left\{p\left(x_{i}\right)\right\}}^{\alpha }$
L(·|·)	The CMIM described as Definition 2
H(·|·)	The conditional Shannon entropy, $H\left(X|Y\right)=\sum_{x_{i}}\sum_{y_{j}}p\left(x_{i},y_{j}\right)\log\frac{1}{p(x_{i}|y_{j})}$
$\Phi_{\varpi }\left(\cdot ||\cdot \right)$	The message importance loss described as Definition 3
*C*	the message importance loss capacity (MILC) described as Definition 4
p(y|x)	An information transfer matrix from the variable *X* to *Y*
{X,p(y|x),Y}	An information transfer process from the variable *X* to *Y*
$\beta_{s}$,$\beta_{e}$,	The parameters in the binary symmetric matrix, binary eraser matrix and
$\beta_{k}$	k-ary symmetric matrix respectively
d(x,y)	The distortion function, d(x,y)≥0
*D*	The allowable distortion ($D_{\min}\leq D\leq D_{\max}$)
$\bar{D}$	The average distortion, $\bar{D}=\sum_{x_{i}}\sum_{y_{j}}p\left(x_{i}\right)p\left(y_{j}|x_{i}\right)d\left(x_{i},y_{j}\right)$
$B_{D}$	The the allowable information transfer matrix set $B_{D}=\left\{q\left(y|x\right):\bar{D}\leq D\right\}$
$R_{\varpi }\left(D\right)$	The message importance distortion function described as Definition 5
I(X||Y)	Mutual information, $I\left(X||Y\right)=\sum_{x_{i}}\sum_{y_{j}}p\left(x_{i},y_{j}\right)\log\frac{p(x_{i},y_{j})}{p\left(x_{i}\right)p\left(y_{j}\right)}$

## Abbreviations

The following abbreviations are used in this manuscript:

MIM	Message Importance Measure
MEMS	Max Entropy in Metric Space
IoT	Internet of Things
NMIM	Non-Parametric MIM
DMIM	Differential MIM
CMIM	Conditional Message Importance Measure
MILC	Message Importance Loss Capacity

## Appendix A. Proof of the Convexity Property of *R_ϖ_* (*D*)

As for an allowable distortion $D_{0}=\delta D_{a}+\left(1-\delta \right)D_{b}$, we have the average distortion for the information transfer matrix $p_{0}\left(y|x\right)=\delta p_{a}\left(y|x\right)+\left(1-\delta \right)p_{b}\left(y|x\right)$ as follows:

(A1) $$\begin{array}{cc} {\bar{D}}_{0} & =\delta \sum_{x_{i}}\sum_{y_{j}}p\left(x_{i}\right)p_{a}\left(y_{j}|x_{i}\right)d\left(x_{i},y_{j}\right)+\left(1-\delta \right)\sum_{x_{i}}\sum_{y_{j}}p\left(x_{i}\right)p_{b}\left(y_{j}|x_{i}\right)d\left(x_{i},y_{j}\right)\\ & \leq \delta D_{a}+\left(1-\delta \right)D_{b}=D_{0}, \end{array}$$

which indicates that the $p_{0}\left(y|x\right)$ is an allowable information transfer matrix for $D_{0}$.

Moreover, by using Jensen’s inequality and Bayes’ theorem, we have the CMIM with respect to $p_{0}\left(y|x\right)$ as follows:

(A2) $$\begin{array}{c} L_{0}\left(\varpi ,X|Y\right)\\ =\sum_{x_{i}}\sum_{y_{i}}p\left(x_{i}\right)p_{0}\left(y_{j}|x_{i}\right)e^{\varpi \{1-\frac{p\left(x_{i}\right)p_{0}\left(y_{j}|x_{i}\right)}{p_{0}\left(y_{j}\right)}\}}\\ =\sum_{x_{i}}\sum_{y_{i}}p\left(x_{i}\right)\left[\delta p_{a}\left(y_{j}|x_{i}\right)+\left(1-\delta \right)p_{b}\left(y_{j}|x_{i}\right)\right]e^{\varpi \{1-\frac{p\left(x_{i}\right)\left[\delta p_{a}\left(y_{j}|x_{i}\right)+\left(1-\delta \right)p_{b}\left(y_{j}|x_{i}\right)\right]}{p_{0}\left(y_{j}\right)}\}}\\ \geq \sum_{x_{i}}\sum_{y_{i}}p\left(x_{i}\right)\left[\delta p_{a}\left(y_{j}|x_{i}\right)\right]e^{\varpi \{1-\frac{p\left(x_{i}\right)\left[\delta p_{a}\left(y_{j}|x_{i}\right)\right]}{p_{0}\left(y_{j}\right)}\}}+\sum_{x_{i}}\sum_{y_{i}}p\left(x_{i}\right)\left[\left(1-\delta \right)p_{b}\left(y_{j}|x_{i}\right)\right]e^{\varpi \{1-\frac{p\left(x_{i}\right)\left[\left(1-\delta \right)p_{b}\left(y_{j}|x_{i}\right)\right]}{p_{0}\left(y_{j}\right)}\}}\\ \geq \delta \sum_{x_{i}}\sum_{y_{i}}p\left(x_{i}\right)p_{a}\left(y_{j}|x_{i}\right)e^{\varpi \{1-\frac{p\left(x_{i}\right)p_{a}\left(y_{j}|x_{i}\right)}{p_{a}\left(y_{j}\right)}\}}+\left(1-\delta \right)\sum_{x_{i}}\sum_{y_{i}}p\left(x_{i}\right)p_{b}\left(y_{j}|x_{i}\right)e^{\varpi \{1-\frac{p\left(x_{i}\right)p_{b}\left(y_{j}|x_{i}\right)}{p_{b}\left(y_{j}\right)}\}}\\ =\delta L_{a}\left(\varpi ,X|Y\right)+\left(1-\delta \right)L_{b}\left(\varpi ,X|Y\right), \end{array}$$

in which

(A3) $$\begin{array}{cc} p_{0}\left(y_{j}\right) & =\sum_{x_{i}}p\left(x_{i}\right)p_{0}\left(y_{j}|x_{i}\right)\\ & =\sum_{x_{i}}p\left(x_{i}\right)\left[\delta p_{a}\left(y_{j}|x_{i}\right)+\left(1-\delta \right)p_{b}\left(y_{j}|x_{i}\right)\right]\\ & =\delta p_{a}\left(y_{j}\right)+\left(1-\delta \right)p_{b}\left(y_{j}\right), \end{array}$$

and the parameter ϖ is $0<\varpi \leq \frac{2\min_{j}\left\{p\left(y_{j}\right)\right\}}{\max_{i}\left\{p\left(x_{i}\right)\right\}}$.

Furthermore, according to the Equations (29) and (A2), it is not difficult to have

(A4) $$\begin{array}{cc} R_{\varpi }\left(D_{0}\right) & =\min_{p\left(y|x\right)\in B_{D_{0}}}\left\{L\left(\varpi ,X\right)-L\left(\varpi ,X|Y\right)\right\}\\ & \leq \{L\left(\varpi ,X\right)-L_{0}\left(\varpi ,X|Y\right)\}\\ & \leq \delta \left\{L\left(\varpi ,X\right)-L_{a}\left(\varpi ,X|Y\right)\right\}+\left(1-\delta \right)\left\{L\left(\varpi ,X\right)-L_{b}\left(\varpi ,X|Y\right)\right\}\\ & =\delta R_{\varpi }\left(D_{a}\right)+R_{\varpi }\left(D_{b}\right), \end{array}$$

where L(ϖ,X) is the MIM for the given information source *X*, while $L_{a}\left(\varpi ,X|Y\right)$ and $L_{b}\left(\varpi ,X|Y\right)$ denote the CMIM with respect to $p_{a}\left(y|x\right)$ and $p_{b}\left(y|x\right)$, respectively.

Therefore, the convexity property is testified.

## Appendix B. Proof of Proposition 4

Considering the fact that the Bernoulli source *X* is given and the equivalent expression is mentioned in Equation (39), the optimization problem $P_{1}$ can be regarded as

(A5) $$\begin{array}{cc} P_{1-A}:\max_{p(y_{j}|x_{i})} & \;\;\;L(\varpi ,X|Y)\\ s.t.\;\;\; & p\left(x_{0}\right)p\left(y_{1}|x_{0}\right)+p\left(x_{1}\right)p\left(y_{0}|x_{1}\right)=D,\\ & p\left(y_{0}|x_{0}\right)+p\left(y_{1}|x_{0}\right)=1,\\ & p\left(y_{0}|x_{1}\right)+p\left(y_{1}|x_{1}\right)=1,\\ & p\left(y_{j}|x_{i}\right)\geq 0,\;\left(i=0,1;j=0,1\right), \end{array}$$

where $L\left(\varpi ,X|Y\right)=\sum_{x_{i},y_{j}}p\left(x_{i},y_{j}\right)e^{\varpi (1-p\left(x_{i}|y_{j}\right))}$ and $0<\varpi \leq \frac{2\min_{j}\left\{p\left(y_{j}\right)\right\}}{\max_{i}\left\{p\left(x_{i}\right)\right\}}$.

To simplify the above one, we have

(A6) $$\begin{array}{cc} P_{1-B}:\max_{\alpha ,\beta } & \;\;\;L_{D}\left(\varpi ,X|Y\right)\\ s.t.\;\;\; & p\alpha +(1-p)\beta =D,\\ & 0\leq \alpha \leq 1,0\leq \beta \leq 1,0\leq p\leq 1, \end{array}$$

in which *p* and (1−p) denote $p(x_{0})$ and $p(x_{1})$, α and β denote $p(y_{1}|x_{0})$ and $p(y_{0}|x_{1})$, and

(A7) $$\begin{array}{c} L_{D}\left(\varpi ,X|Y\right)\\ =p\left(1-\alpha \right)e^{\frac{\varpi (1-p)\beta }{p(1-\alpha )+(1-p)\beta }}+\left(1-p\right)\beta e^{\frac{\varpi p(1-\alpha )}{\left(1-p)\beta +p(1-\alpha \right)}}+\left(1-p\right)\left(1-\beta \right)e^{\frac{\varpi p\alpha }{p\alpha +(1-p)(1-\beta )}}+p\alpha e^{\frac{\varpi (1-p)(1-\beta )}{p\alpha +(1-p)(1-\beta )}}, \end{array}$$

where $0<\varpi \leq \frac{2\min_{j}\left\{p\left(y_{j}\right)\right\}}{\max_{i}\left\{p\left(x_{i}\right)\right\}}$.

Actually, it is not easy to deal with the Equation (A6) directly; we intend to use an equivalent expression to describe this objective. By using Taylor series expansion of $e^{x}$, namely $e^{x}=1+x+\frac{x^{2}}{2}+o\left(x^{2}\right)$, we have

(A8) $$\begin{array}{cc} L_{D}\left(\varpi ,X|Y\right) & ≐1+\left(2\varpi +\frac{\varpi^{2}}{2}\right)\left\{\frac{p\alpha (1-p)(1-\beta )}{p\alpha +(1-p)(1-\beta )}+\frac{p(1-\alpha )(1-p)\beta }{p(1-\alpha )+(1-p)\beta }\right\}. \end{array}$$

By substituting $\beta =\frac{D-p\alpha }{1-p}$ into the Equation (A8), it is easy to have

(A9) $$\begin{array}{cc} L_{D}\left(\varpi ,X|Y\right) & ≐1+p\left(2\varpi +\frac{\varpi^{2}}{2}\right)\left\{\frac{p\alpha^{2}+\left(1-p-D\right)\alpha }{2p\alpha +(1-p-D)}+\frac{p\alpha^{2}-\left(p+D\right)\alpha +D}{(p+D)-2p\alpha }\right\}, \end{array}$$

where $\max\left\{0,1+\frac{D-1}{p}\right\}\leq \alpha \leq \min\left\{1,\frac{D}{p}\right\}$ resulted from the constraints in Equation (A6).

Moreover, it is not difficult to have the partial derivative of $L_{D}\left(\varpi ,X|Y\right)$ in Equation (A9) with respect to α as follows:

(A10) $$\begin{array}{cc} \frac{\partial L_{D}\left(\varpi ,X|Y\right)}{\partial \alpha } & ≐2p^{2}\left(2\varpi +\frac{\varpi^{2}}{2}\right)\left\{\frac{-p\alpha^{2}-\left(1-p-D\right)\alpha }{{\left[2p\alpha +\left(1-p-D\right)\right]}^{2}}+\frac{p\alpha^{2}-\left(p+D\right)\alpha +D}{{\left[\left(p+D\right)-2p\alpha \right]}^{2}}\right\}. \end{array}$$

By setting $\frac{\partial L_{D}\left(\varpi ,X|Y\right)}{\partial \alpha }=0$, it is readily seen that the solutions of α in Equation (A10) are given by $\alpha_{1}=\frac{(1-p-D)D}{p(1-2D)}$ and $\alpha_{2}=\frac{1-D-p}{1-2p},$ respectively.

In addition, in the light of the domain of *D* mentioned in Equation (35), it is easy to have $D_{\max}=\min\left\{p,1-p\right\}$ in the Bernoulli source case. That is, the allowable distortion satisfies 0≤D≤min{p,1−p}. Thus, the domain of α namely $\max\left\{0,1+\frac{D-1}{p}\right\}\leq \alpha \leq \min\left\{1,\frac{D}{p}\right\}$, can be given by $0\leq \alpha \leq \frac{D}{p}$.

Then, it is easy to have the appropriate solution of α as follows:

(A11) $$\begin{array}{c} \alpha^{*}=\frac{(1-p-D)D}{p(1-2D)}, \end{array}$$

in which the second derivative $\frac{\partial^{2}L_{D}\left(\varpi ,X|Y\right)}{\partial \alpha^{2}}$ is non-positive, namely maximum value is reached, and the corresponding information transfer matrix is

(A12) $$\begin{array}{c} p\left(y|x\right)=\left[\begin{array}{cc} \frac{\left(1-D)(p-D\right)}{p(1-2D)} & \frac{(1-p-D)D}{p(1-2D)}\\ \frac{D(p-D)}{\left(1-p)(1-2D\right)} & \frac{\left(1-p-D)(1-D\right)}{\left(1-p)(1-2D\right)} \end{array}\right], \end{array}$$

where 0≤D≤min{p,1−p}.

Consequently, by substituting the matrix Equation (A12) into the Equation (40), it is not difficult to verify this proposition.
